# General practitioners’ educational and training needs and requirements for advising patients with coronary heart disease on physical activity: findings from a qualitative study in Germany

**DOI:** 10.1186/s12875-025-02973-0

**Published:** 2025-08-29

**Authors:** Alicia Prinz, Sabrina Hoppe, Rik Crutzen, Stefan Wilm, Sabrina Kastaun

**Affiliations:** 1 Institute of General Practice (ifam), Patient-Physician-Communication Research Unit, Centre for Health and Society (chs), Medical Faculty and University Hospital, Heinrich-Heine-University Düsseldorf, P.O. Box 101007, Düsseldorf, 40001 Germany; 2 Department of Health Promotion, Care and Public Health Research Institute (CAPHRI), Maastricht University, Maastricht, The Netherlands

**Keywords:** Chronic ischemic heart disease, Coronary heart disease, Exercise, General practice, Primary care, Qualitative research, Brief counselling, Guidance, Education, Training

## Abstract

**Background:**

The World Health Organization and German treatment guidelines emphasise integrating physical activity (PA) advice into primary care of patients with coronary heart disease (CHD). However, this is often inadequately implemented in German general practice. A key barrier may be healthcare professionals’ limited knowledge and skills in providing effective and efficient PA guidance – both in general as well as for patients with CHD. International guidelines recommend targeted training for general practitioners (GPs). Understanding GPs’ specific educational and training needs is crucial to developing tailored training programmes for improving implementation of advising CHD patients on PA.

**Methods:**

Between March and June 2023, 12 problem-centred one-on-one interviews and six moderated focus groups (*n* = 37 participants) with GPs were conducted in-person (total *N * = 49 GPs, 37% female, mean age 56 years (36–78 years)). Interview and focus group topic guides were developed and pilot-tested by the multi-professional study team. Transcripts from audio-recorded data were analysed using both deductive and inductive content structuring, within a multi-professional team, with active involvement of GPs. This analysis extends a previous study using the same dataset.

**Results:**

GPs recognise the benefits of PA for CHD patients. Their conceptualisations of PA (e.g., definitions of sport and PA, general statements on PA) vary and the incorporation of PA in everyday activities (e.g., using stairs) to encourage behaviour change is sometimes recommended. GPs identify the following key training needs for providing PA advice to CHD patients: peer exchange and self-reflection, evidence-based knowledge on PA and CHD, practical tools and support materials to facilitate the integration of PA advice into routine practice. These include low-barrier, time-efficient communication techniques, and role-play simulations.

**Conclusion:**

This qualitative study provides insights into GPs’ needs and preferences regarding the content, didactics, and organisation of training on PA advice. To enhance competence and foster deeper learning, training should combine evidence-based knowledge with practical elements such as simulations and reflective peer exchange. Building on previous findings about GPs’ attitudes and experiences, the identified educational needs offer a foundation for developing tailored training to support PA counselling in routine primary CHD care. This training will be implemented and evaluated in a subsequent study.

**Supplementary Information:**

The online version contains supplementary material available at 10.1186/s12875-025-02973-0.

## Background

Chronic coronary heart disease (CHD), a major global contributor to morbidity and mortality [1], affects approximately 9% of adults aged 40 to 79 years in Germany [2]. Patients with stable CHD are primarily treated in general practice, often within the framework of structured Disease Management Programmes (DMPs).

In managing CHD, regular physical activity (PA) is an effective measure to reduce the risk of myocardial infarction, lower cardiovascular mortality rates and all-cause hospitalisations, and to improve health-related quality of life in affected individuals [3, 4].

However, physical inactivity is particularly prevalent among individuals with CHD. In Germany, only around 39% of adults with CHD meet the recommendations of the World Health Organization (WHO) for aerobic PA of at least 150 min per week [5]. This highlights the need for tailored support for this group – similar to other individuals living with non-communicable chronic diseases [5].

Advice on PA provided by general practitioners (GPs) – who are seen as trusted advisors on health behaviour by their patients [6] – can increase the chance for PA behaviour change in those receiving advice and support [7–10]. A systematic review specifically focusing on brief interventions [11] revealed that brief advice by GPs can improve patients’ self-reported and measured PA levels, at least in the medium term (up to six months). Consequently, the WHO [12], international treatment guidelines [13] and the German clinical treatment guideline on CHD [14] all recommend that health professionals (HPs), including GPs, should offer advice on PA to individuals with CHD.

However, PA advice from GPs appears to be inadequately integrated into German primary care [15, 16]. International qualitative studies on GPs’ experiences with lifestyle counselling in routine care [17–22], particularly focussing on PA [23–27], and those addressing other conditions than cardiovascular diseases – primarily diabetes [25] or obesity [28] –, have identified several key barriers to the routine delivery of PA advice to patients. These barriers include competing demands in daily practice, challenges in providing individualised PA advice, difficulties in assessing the PA status of patients or providing concrete information on PA opportunities. Almost all studies highlight the need of (further) education and training for HPs in how to effectively and efficiently advise patients on PA behaviour, and emphasise the importance of customised training approaches. Such training can be even more important for supporting patients living with chronic illnessess such as CHD and addressing their specific needs [19, 27, 29–32]. 

Appropriate training for HPs, including GPs, is also recommended by international guidelines [13, 33]. However, educational approaches on advising and supporting patients to improve their health behaviour are neither part of the medical curricula nor of vocational or continuing medical education in Germany.

In order to support GPs in providing their patients with low-threshold PA advice – that is, brief, time-efficient guidance that can be easily integrated into routine consultations without overwhelming patients – training should be tailored to GPs’ specific educational needs and preferences. Such brief and effective PA advice may contribute to improving preventive care for patients with CHD. To develop such tailored training programmes for GPs in advising CHD patients on PA, in-depth information is needed on GPs’ experiences, perceptions, and attitudes regarding PA advice in everyday care [17–22]. It is also essential to understand GPs’ specific requirements and needs for appropriate education and training.

In a previous analysis [34], based on the same dataset, we explored GPs’ experiences, perceptions, and attitudes toward providing PA advice to patients with CHD, using qualitative interviews and focus groups. Similar to findings from international studies highlighting the importance of training HPs in PA counselling [19, 29, 30, 35], the participating GPs in our previous analysis emphasised the need for practical skills and CHD-specific knowledge and PA advice [34].

Both the previous analysis and the current analysis draw on the same qualitative dataset and are one substudy of the overarching OptiCor project (“Optimising the treatment of chronic ischemic heart disease by training general practitioners to deliver very brief advice on physical activity”) [34]. The OptiCor project aims to systematically develop and evaluate a tailored training to support GPs in the effective delivery of PA advice during routine care of patients with CHD.

To our knowledge, no German studies have yet explored whether GPs are interested in training on PA advice, nor their requirements regarding content, teaching methods, organisation, and perceived needs required to deliver such advice effectively. These insights can inform the development of a training for GPs by emphasising clear, practical knowledge and skills to enhance PA counselling. Such training has the potential to positively influence GPs’ practices in advising patients with CHD.

## Methods

This study is reported in accordance with the consolidated criteria for reporting qualitative research (COREQ) [36]. The literature search for this manuscript (e.g., for the sections background and discussion) followed a structured approach, including the use of search strings (e.g., in PubMed) and a review of relevant guidelines and projects.

### Study design

We conducted a qualitative study using problem-centred one-on-one interviews [37] and moderated focus groups [38, 39] with GPs, and analysed them using a content structuring procedure (deductive-inductive approach) [40]. In order to gain the deepest and broadest perspective as possible we decided to combine interviews and focus groups. While sensitive and personal revelations often occur in individual interviews without the influence of group dynamics, group dynamics in focus groups can help to reveal and reconstruct collective orientations, and often provide a deeper insight into collective attitudes, prejudices and problem-solving strategies [39].

### Recruitment and sampling

Using a purposive sampling [41], we invited 80 GPs in the German federal state of North-Rhine-Westphalia (NRW) through email or by personal invitation to participate in individual interviews. For focus groups, 62 regional GP networks were invited to participate.

In total, 20 GPs were interested in participating in interviews and seven regional GP networks were interested in participating in focus groups. Inclusion criteria were as follows: currently practicing in general practice, general internal ambulatory medicine, or specialising in one of these fields. Participants also needed sufficient German language skills and had to sign an informed consent form before study inclusion.

To ensure a broad sample of GPs, a brief questionnaire was used as part of the sampling process and the selection of GPs, conducted via telephone before each GPs’ inclusion in the study. This questionnaire is available as supplementary file (Appendix 1, in English) or at Open Science Framework (OSF, https://osf.io/yj9dr, in German). Because entire existing GP networks were targeted for recruitment, it was not possible to apply specific sampling strategies at the individual GP level for participants of the focus groups.

Characteristics of the resulting 49 GPs who participated in either an interview or a focus group (12 interviews, six focus groups with a total of 37 GPs) are presented in Table 1. Characteristics of the study sample have already been published in the first part of the qualitative study, and are therefore presented in the method section of this manuscript instead of the result section [34]. 

**Table 1:** Characteristics of interviewed general practitioners who participated in either an interview or a focus group

	Interviews (*n* = 12)	Focus groups (*n* = 37)	Total (*N* = 49)
Sex			
Male	7	24	31
Female	5	13	18
Age, mean (min.-max. years)	55.4 (35–73)	56.3 (36–70)	56.1 (35–73)
Migration background			
Yes	1	10	11
No	10	27	37
Main medical speciality			
General practice/family medicine	7	22	29
General internal ambulatory medicine	4	12	16
Doctor in further specialty training	1	0	1
Other	0	3	3
Years actively working as a GP, mean (min.-max. years)	17.0 (1–38)	19.4 (1–36)	18.8 (1–38)
Type of practice			
Single handed practice without physician employees	3	15	18
Individual practice with physician employees	5	5	10
Joint/group practice	4	17	21
Focus on sports medicine			
Yes	1	6	7
No	11	31	42
Academic teaching practice			
Yes	11	14	25
No	1	23	24
Patients per quarter of a year			
< 1000	1	6	7
1000–2000	6	15	21
> 2000	5	16	21
Location of the practice			
Rather rural area	3	19	22
Small/medium-sized town	6	11	17
Urban area	3	7	10

Missing data per variable (age: *n* = 1, migration background: *n* = 1)

### Data collection

We have asked participating GPs to envision training on advising patients with CHD about PA. We encouraged them to reflect on their specific needs in terms of content, didactics and organisation, as well as their personal requirements for improving their PA counselling skills. In addition, we aimed to assess GPs’ existing knowledge about PA, its role in CHD, and GPs’ conceptual understanding of PA. The interview and focus group topic guides aimed to address our research aims and were developed and pilot tested by the multi-professional study team comprising GPs and researchers with different backgrounds in psychology, sociology, and public health. Tables 2 and 3 provide an overview of the themes and narrative questions of the interview and focus group topic guides. The full topic guides are available as supplementary files (Appendix 2, Appendix 3, both in English) or in German at OSF: https://osf.io/yj9dr.

**Table 2:** Brief overview of the themes and narrative questions covered in the interview guide

Theme	Narrative question
1. Experiences with advice on physical activity in the context of CHD	Do you remember your last patient with coronary heart disease in your practice? Can you walk me through that consultation? Please describe in as much detail as possible how you experienced it.
	And how was it with those patients when it came to the topic of physical activity? Could you tell me more about that?
2. Personal attitudes and motivators for physical activity advice	In your role as a GP, what do you see as the most important things you can do for patients with stable CHD?
	When you talk with patients who have CHD, what topics are important for you to cover?
	What are your personal thoughts about physical activity for people with CHD?
	So far, we’ve talked about your experiences and priorities when it comes to these conversations. Now I’d like to ask: how does this play out in your daily practice? What’s your experience with this in everyday routines?
3. Requirements for a training programme	Now that we’re talking about training – I’d like you to imagine a training for GPs on advising patients with CHD about physical activity. What comes to your mind?
	Can you recall a training (or several) that you left feeling truly inspired? Feel free to tell me more about it.
4. Final thoughts and anything left unsay	We’re now at the final question. Thank you – your insights have been incredibly valuable. If you could wish for three things related to what we’ve discussed in the past hour – what would they be?

The present study mainly analyses the content concerning Theme 3

**Table 3:** Brief overview of the themes and narrative questions covered in the focus group topic guide

Theme	Narrative question
1. Opening phase and welcome	
2. Needs and requirements for advice on physical activity in CHD care	You care for patients with coronary heart disease in your practice. When you talk to these patients, what do you usually discuss? Try to recall a recent consultation – maybe from the past few days or weeks – perhaps within the CHD Disease Management Programme.
3. Requirements for a training programme	I’d like to invite you to imagine a training session on how to talk with patients about physical activity. What would you need for it to be helpful? What comes to your mind?
	To encourage further input: What would the training need to include for you to leave feeling equipped and motivated to hold effective and enjoyable conversations about physical activity the next day?
4. Final question	At the end of this training on advising patients about physical activity – what would need to happen during that session for you to feel ready and motivated to go back and have good conversations with your CHD patients the next day?
5. Closing phase and farewell	

The present study mainly analyses the content concerning Theme 3

Between March and May 2023 the main author (female, research associate and sociologist with qualitative research and interview experience) conducted all twelve interviews in person, primarily at the GPs’ practices. Interview lengths ranged from 34 to 71 min, with an average duration of 54 min.

Six focus groups, each involving five to ten participants (*n* = 37 GPs in total), were conducted in person between April and June 2023 by three experienced group moderators from the study institute (all female, including a GP, the main author and a psychotherapist with longstanding research experience in general practice). An accompanying study team member (all female, research associates) took notes to assist speakers’ identification for transcription. With one exception, all focus groups were held in the usual locations of the GPs network meetings. Five of the six focus groups consisted of participants who were mostly familiar with each other, as they regularly attended network meetings on GP-related topics. Notes taken by the study team during focus groups indicated a predominantly trusting and relaxed atmosphere. In contrast, one focus group was conducted during an event hosted by the study institute, and most participants did not know each other beforehand. Focus groups lasted between 72 and 92 min, with an average duration of 84 min.

No interviews or focus groups were repeated. Field notes documented the atmosphere, conversation flow, unique aspects, and conversation disruptions. All interviews and group discussions were audio-recorded and transcribed verbatim. No professional or personal relationships existed between researchers and participants. Participating GPs received an incentive of €50.

### Data analysis

We analysed the data using a content structuring approach, which is a category based, language related and rule guided scientific method designed to systematically reduce complexity [40]. Using both deductive and inductive techniques, we developed a coding guide and defined all categories in a category manual within the multi-professional study team (see above). All interviews and focus groups were coded concurrently by two researchers from a team of six coders over a period of six months. To incorporate a broad range of perspectives, feedback loops were conducted within the study team, e.g., to discuss adjustments to the coding guide. To ensure the quality of the process, we conducted two multi-professional analysis groups (6–7 participants each, including e.g., GPs and researchers with backgrounds in psychology, sociology, public health, and sport and exercise gerontology). GPs were continuously involved throughout the research process, e.g., in the creation of topic guides for interviews and focus groups, in the coding of data material and in analysis groups. Findings were revalidated in a separate workshop with GPs. All data were managed using the software MAXQDA [42]. 

The analysis was conducted using the original German transcripts. Quotations cited in the manuscript were later translated into English and reviewed by two additional researchers. As with any translation, it is important to note that cultural and regional language nuances may influence interpretation.

## Results

Using deductive and inductive categorisation techniques, we developed 13 main thematic categories (overarching themes), which were further differentiated into 28 subcategories (sub-themes). The complete coding tree has been published at OSF (https://osf.io/yj9dr, translated into English).

**Fig. 1:**
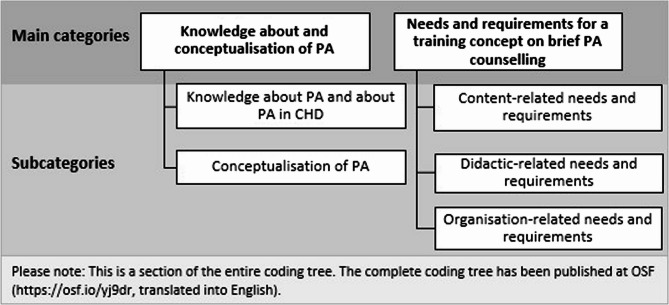
Central main categories and their subcategories derived from interviews and focus groups with GPs (*N* = 49) as part of the qualitative content analysis

While a previous analysis [34] focussed on GPs’ experiences, perceptions and attitudes regarding PA advice for patients with CHD, the current analysis addresses GPs specific needs and requirements regarding training content, didactics, and organisation, as well as their knowledge and conceptual understanding of PA in the context of CHD. In line with these objectives, we focus our presentation of results on two thematic categories ‘knowledge about and conceptualisation of PA’ and ‘needs and requirements for a training concept’ (Fig. 1). 

Typical original quotations from GP interviews and focus groups translated into English are presented in Tables 4 and 5 to support the summarised results.

**Table 4:** Quotations of the main category ‘knowledge about and conceptualisation of PA’ organised by subcategories (translated from German into English^a^)

Subcategory	Quote No.	Quotation
Knowledge about PA and about PA in CHD	1a	‘You not only get a benefit in terms of your coronary heart disease, but you also lose weight, your metabolism improves, your blood pressure regulates itself, your risk of cancer minimises, you enhance your performance and your quality of life - it’s a colourful bouquet of positive things.’ (HA_FG_03, GP A)
	1b	‘If we know which four risk factor constellations are the ones that influence our morbidity the most, that is nicotine, alcohol, lack of physical activity, and malnutrition.’ (HA_FG_02, GP F)
	1c	‘I would also find that interesting: are there any figures on how much physical activity minimises the risk, how much physical activity do I need?’ (HA_FG_01, GP A)
Conceptualisation of PA	2a	‘I mean, they don’t need to become top athletes, but physical activity is important. It’s really not just young people, but also older individuals who need to stay active, not just sit in front of the TV and become sedentary. The goal is to improve their quality of life.’ (HA_Int_01)
	2b	‘What I emphasize here is that it’s not about doing high-performance sport, but about saying that any kind of physical activity is good, e.g. walking the dog is just as effective as cycling or swimming, so we should take the focus off performance[…] and instead highlight the opportunities for everyday activity.’ (HA_Int_08)
	2c	‘If you then engage in sports, you have to do endurance training, otherwise it won’t be as effective.’ (HA_Int_02)
	2d	‘In discussions with CHD patients, there is of course also the age limit, yes? From a certain age, sport is simply no longer plausible for them, right? It´s no longer part of it.’ (HA_FG_05, GP A)

^a^The analysis of the study is based on the original German transcripts. Quotations were subsequently translated and reviewed by two other researchers. However, every translation is also an interpretation, due to cultural and regional specific language

**Table 5:** Quotations of the main category ‘requirements and needs for training concept’ organised by subcategories (translated from German into English^a^)

Subcategory	Quote No.	Quotation
Content-related	1a	‘That you simply enter into a discussion with colleagues based on a specific topic or a keynote speech and then openly exchange experiences: Does it help? What experiences have you had with it? These are actually the events where you’re most likely to take something away afterwards or feel validated in the way you do things.’ (HA_Int_09)
	1b	‘How do we actually manage to talk about the potential for change? This involves self-motivation, external motivation, and incentives. And for me, this is closely linked to the topic of physical activity. And if you ask me, that´s actually part of it. What actually prevents us, what makes us resign ourselves in advising or with patients?’ (HA_FG_05, GP C)
	1c	‘What does physical activity mean to me? Why do I think my patients don’t exercise? Why don’t I address this?’ (HA_Int_08)
	1d	‘What impact does physical activity have compared to, for example, statin therapy in patients who already have CHD? Having a good data basis for this.’ (HA_Int_08)
	1e	‘What I would find helpful is clear information on the topic: What is evidence-based with regard to CHD and physical activity? What is supported by reliable data that you can give people in a reproducible and reliable way?’ (HA_Int_12)
	1f	‘How can I find out from the patient what they do and what they don’t do? […] How can I find out where they actually stand through dialogue or a brief intervention?’ (HA_FG_05, GP A)
	1 g	‘It’s communication training. It´s about improving communication so that I can motivate the patient and this may also involve experiencing positive examples of inner attitudes towards the patient so that the conversation is successful and the patient is then able to implement physical activity.’ (HA_Int_07)
	1 h	‘It’s about talking about, seeing, and hearing what colleagues say or advise in certain situations. It’s not about acquiring new cognitive knowledge. I think that’s obvious, everyone knows it, everyone has it at hand.’ (HA_Int_10)
	1i	‘So, the training sessions that I benefited from also involved roleplays […] of course that’s a super uncomfortable situation, you don’t really want to be in it, but it’s incredibly useful […] when you’re sitting there uninvolved and then: Hmm, why isn’t the colleague engaging with this? But I also found it very helpful to see how certain things caused a reaction and to really experience that. I was able to take a lot with me back then, I don´t want to say that I always manage it, but I really took a few key things with me and found it extremely helpful.’ (HA_FG_04, GP B)
	1j	‘What would appeal to me would be exercise programmes that are easy to implement, simple tips on that. There are also sports programmes that are not very well-known, but which would be good to find out more about what options are available.’ (HA_Int_06)
Didactic-related	2a	‘It’s great to be at a new level here. Especially, as I said, whenever it´s suitable for everyday use, when I think: I can put it into practice like this.’ (HA_Int_04)
	2b	‘An exercise program, a kind of take-away effect, something that is exciting for you, so that you don´t just learn: What can I do for other people? But also gaining something good for yourself. For me personally, for example, it would be great to have something like yoga in the evening, I think that would be nice. If you could combine that with a practical part and do something like that.’ (HA_Int_08)
	2c	‘What I would really be interested in is: What do they do in the cardiac exercise groups, and what does it look like? What do they do right, and what might they be doing wrong? It would be helpful to actually experience it, maybe even taking part in a session yourself. Then you realise what you can recommend to patients and what not.’ (HA_Int_12)
	2d	‘Also, to talk to sports scientists or something, so it’s not just one-sided from the medical perspective, but also includes input from other areas, from physiotherapy, for example.’ (HA_Int_01)
Organisation-related	3a	‘But apart from that, I very rarely go to training courses. So I do it in writing at home. I: What exactly stops you from doing that? P: [exhales] Very practically: I have a wife who works full time three children at home.’ (HA_Int_11)
	3b	‘So not an additional event on top of the existing DMP obligations, because that often requires a lot of time and effort anyway. But within the framework of these training course, it would be good to say: Okay, this is a simple tool for the care of CHD patients, because it is inexpensive, low-effort and can be customised.’ (HA_Int_08)

^a^The analysis of the study is based on the original German transcripts. Quotations were subsequently translated and reviewed by two other researchers. However, every translation is also an interpretation, due to cultural and regional specific language

### Knowledge about and conceptualisation of PA

#### Knowledge about PA and about PA in CHD

In this category, we describe implicit knowledge and assumptions derived from statements made by the participating GPs, which reflect their knowledge about PA in the context of patient care and specifically in the context of CHD. This includes recollections of knowledge, statements regarding the evidence of PA and PA in CHD, as well as references to gaps in knowledge and misconceptions.

Most participating GPs had a general understanding of the benefits of PA, including its beneficial role in reducing cancer risk, stress and pain and enhancing the quality of life and cognitive health (quote 1a, Table 4). A few GPs identified physical inactivity as a risk factor for the development of non-communicable diseases (quote 1b, Table 4).

While the majority of GPs was aware of the benefits of PA, insights into the specific evidence supporting PA in the management of CHD varied, with some GPs questioning its effectiveness in this regard. Gaps in knowledge included uncertainties about the specific effects of PA on the progression of CHD, recommended frequency and intensity of PA to balance avoiding overexertion in patients while achieving benefits for their heart disease, and specific PA recommendations, e.g., sports types (quote 1c, Table 4).

#### Conceptualisation of PA

The results of this category cover PA conceptualisations of the participating GPs in general patient care and in the context of CHD, e.g., definitions of sport and PA, general statements on PA, on types of PA, or statements on persons and groups that indicate GPs’ understanding of PA.

GPs had varying conceptualisations of PA in the context of preventive medicine. Some GPs associated PA with different intensities or certain activities as some of their recommendations given to patients focus on performance-oriented or institutionalised approaches to PA, e.g., cardiac sports groups (quote 2a and c, Table 4). For some GPs, the primary goal was to encourage patients to become more active than before or at least active at all, whereas others focused on the importance of endurance training to achieve certain health effects (quote 2b and c, Table 4).

The majority of participating GPs seemed to believe that PA is suitable for everyone, regardless of factors like age. However, some of the GPs struggled to imagine individuals with limited mobility being physically active. In this context, PA is rather recommended to younger patients without physical restrictions (quote 2 d, Table 4).

Across the different PA conceptualisations, incorporating everyday activity (e.g., taking the stairs instead of the lift) and activities that bring joy to improve PA behaviour was sometimes mentioned. In this regard, GPs discussed the advantages of everyday activities, such as their low-threshold accessibility (e.g., when going for a walk or walking the dog) and the potential for these PA activities of becoming a habit. This low-threshold approach is also used to recommend PA to patients facing barriers – such as financial limitations – which makes it difficult to access external PA offers. In this context, many of the GPs were aware of and sensitive to diverse life situations of their patients.

### Needs and requirements for a training concept on brief PA counselling

#### Content-related needs and requirements

Nearly all participating GPs emphasised the need for peer exchange on advising patients about PA. Such exchange was valued for providing mutual support on daily practice, including assistance with expressing professional concern and interest about patients’ health effectively, and enhancing skills in motivational interviewing (quote 1a, Table 5).

Some GPs also highlighted the value of guided self-reflection, e.g., through a peer trainer. Identified areas for reflection included the role and attitudes of GPs towards providing PA advice, the importance of PA advice by GPs, the omission of PA discussions in patient consultations, target groups and individuals who receive less advice (and the reasons for this), and GPs own PA behaviour (quote 1b and c, Table 5).

The majority of participating GPs mentioned a strong need for information on evidence-based knowledge on health-beneficial effects of PA in general, in patients with CHD (e.g., compared to drug therapy), on specific PA recommendations for CHD (e.g., frequency, intensity), and on the effectiveness of GP advice on PA (quote 1 d and e, Table 5). Few GPs asked for instructions on how to assess a patient’s PA history and current PA behaviour (quote 1f, Table 5).

Low-threshold and timesaving communication techniques for PA advice were also required from some of the participating GPs as a central aspect of a training on PA advice (e.g., conversation guides, motivational interviewing, brief advice approaches) (quote 1 g, Table 5). In this regard participating GPs also noted specific concerns, such as overly structured discussions that may not align with empathetic and patient-centred conversations between GPs and patients, particularly in the context of long-standing patient-physician relationships. In contrast, few of the participating GPs did not express a need for further knowledge on beneficial effects of PA in CHD or on advising patients on PA. In these cases, sufficient knowledge or a higher priority on peer exchange were given as reasons (quote 1 h, Table 5).

Some participating GPs expressed the need for support materials which could be used during conversations with their patients on PA or to be passed on to patients, including visualisations of evidence-based knowledge on the effectiveness of PA in CHD, PA recommendations on frequency and intensity, patient-friendly information material on specific and low-threshold (digital) PA recommendations, a compilation of regional PA programmes for individuals with CHD, and information on reimbursement options or financial subsidies for PA programmes (e.g., from health insurance companies) (quote 1e and j, Table 5).

#### Didactic-related needs and requirements

The participating GPs agreed that all training content should be low-threshold, practical, directly implementable, and tailored to their specific needs (quote 2a, Table 5). Interactive training formats were seen as particularly beneficial, with elements that support these formats – such as small group sizes and a trusting atmosphere – being highly valued.

Role plays with simulation patients (brought in by the participating GPs) were seen as effective by few participating GPs when teaching communication techniques, but were largely rejected by most of the participating GPs. A main reason seems to be that acting in front of peers is perceived as intimidating and unpleasant (quote 1i, Table 5).

Notably, GPs expressed interest in incorporating PA into the training itself, such as through PA breaks. They suggested that experiencing the immediate effects of PA could enhance their self-awareness and motivation to provide PA advice. Additionally, some GPs proposed exercises from rehabilitation or cardiac sports programmes that could be directly recommended to patients (quote 2b and c, Table 5).

Very few GPs expressed a need for an interdisciplinary or interprofessional training approach. Their suggestions ranged from including sports science or physical therapy expertise to designing a multiprofessional training programme involving other HPs, such as medical assistants or physiotherapists (quote 2 d, Table 5).

#### Organisation-related needs and requirements

The preferred training format for GPs varied including online, blended learning, or face-to-face formats, and seemed to be strongly influenced by their experiences with previous training and personal factors, such as commitments to their practice or family, or long travel distances to the training location from more rural areas. However, for the majority of GPs, the format of the training did not seem to be a determining factor for their final participation (quote 3a, Table 5).

Most GPs welcomed the integration of a training on advising CHD patients on PA into existing formats, such as mandatory vocational or continuing medical education in disease management programmes or group discussion meetings within GP networks (quote 3b, Table 5).

## Discussion

### Principal findings

This study examines German GPs’ needs regarding content, organisation and didactics of a PA training, as well as their knowledge and conceptualisation of PA in CHD. Combined with earlier findings [34], it aims to inform the design of a targeted GP training for delivering effective and efficient PA advice during routine CHD consultations.

Most GPs demonstrate general awareness of PA’s health benefits (e.g., improved quality of life, cancer risk reduction), yet report limited knowledge of specific evidence-based PA recommendations tailored to CHD patients, including type, intensity, and clinical efficacy. PA is not consistently framed within preventive medicine.

While some GPs encourage everyday PA – a flexible and evidence-supported approach – this is not widely practiced. Others lean toward performance-oriented or institutionalised PA offers, such as cardiac sports groups. The findings underscore the importance of emphasising everyday activity promotion, reinforcing that ‘every step or minute counts’ [43, 44].

Our findings reveal a strong desire for peer exchange and reflective discussion on PA advice in CHD care. Some GPs expressed uncertainty about how to deliver effective and efficient PA counselling. Peer-supported dialogue, incorporating reflective questioning and practical methods, may help build confidence and validate approaches.

Participants clearly articulated preferences for training that is low-threshold, practice-oriented, and directly applicable to their clinical routines, supported by material useful for both clinicians and patients. Practical relevance and alignment with the specific context of GP care – particularly the continuity of patient relationships – is essential for training acceptance.

### Comparison with existing literature

GPs’ lack of education and training may contribute to gaps in knowledge and skills, as well as to unsatisfactory experiences in providing PA advice to patients in general [34, 45, 46]. However, to the best of our knowledge, there are no qualitative studies in Germany that have investigated in depth the knowledge and conceptual understanding of PA in the context of CHD patient care among GPs, as well as the content, organisational, and didactic requirements for a GP training on PA advice.

Some quantitative studies have examined GPs’ knowledge and competence regarding various health aspects, including the frequency of PA advice [46, 47]. For example, a German study about the promotion of PA among older adults found that physicians (*N* = 60, including 48 GPs) rated their knowledge about PA and their skills in initiating and advising on PA at an intermediate level [47]. A study from the United Kingdom concluded that 80% of GPs were unfamiliar with the national PA guidelines [46]. 

Our findings on knowledge partially align with those of a qualitative study conducted in Ireland, which used semi-structured online interviews with HPs to analyse the integration of PA in routine practice with older adults [48]. Although the majority of the 63 participants were not GPs (only 16%) the participants recognised the general benefit of PA for older adults and demonstrated knowledge of specific areas, such as fall prevention [48]. Despite methodological differences to our study, the Irish study’s insights into the support HPs need to deliver effective and efficient PA advice are consistent with our results. The HPs interviewed expressed the need for tailored educational opportunities, emphasising brief, practical training sessions grounded on real-life scenarios and simulated GP consultations [48]. 

Although research on HPs knowledge of PA and its role in other health conditions is limited, a few quantitative studies can be found regarding specific conditions such as cancer [49], and with focus on oncologists and oncology health care providers [50]. A systematic review (survey-based) reported that HPs have limited knowledge of PA guidelines for cancer patients [51]. 

Parallels can also be drawn with the above mentioned German questionnaire survey in physicians, where they were asked about preferred types of information and training formats on PA advice [47]. The majority of participants (*n* = 38/60) favoured a short seminar, while 27 participants expressed interest in a brief self-administered intervention, and 37 participants were interested in patient materials for older adults [47]. Participants in another German qualitative study about health promotion in primary care – without allocation to a specific target group – highlighted the preference for digitally accessible, innovatively designed informational materials, along with an overview of where to find information on specific topics [52]. 

Other studies from the United Kingdom, Canada and Finland have proposed training concepts for advising patients on PA without reference to specific diseases [46, 53, 54]. In most cases, the training components – such as assessing patients’ PA level, using motivational interviewing techniques to promote PA, and providing written PA instructions – are derived from quantitative studies [53]. These studies underscore a lack of training, knowledge, or tools for prescribing PA [46]. However, it remains unclear to what extent the developed training programmes are grounded in the findings of these studies [55]. 

Notably, some components of these developed trainings overlap with those mentioned by the GPs we interviewed, such as the peer-to-peer approach [56]. This highlights the importance of integrating evidence-based strategies with practical, context-specific elements when designing training programmes for PA advice.

### Strengths and weaknesses of the study

The continuous involvement of the target group in the development of interview and focus group topic guides, analysis and interpretation of the data is the main strength of this qualitative study. Results were thus consistently reviewed and validated from individuals with different perspectives. In addition, all data were double-coded (primary and control), which contributes to the quality of the coding process and findings following from those.

The study also has limitations. GPs who agreed to participate might more likely have an interest in providing lifestyle and PA advice, which may have motivated them to share their experiences. Furthermore, their personal interests could have also influenced their shared experiences. In order to still capture a diverse range of GPs and their perspectives (e.g., in terms of professional experience, practice location, and interest in PA), and to minimise the potential selection bias, we used multiple sampling strategies: For focus groups, we aimed to include all GPs of a GPs network, which may have increased the chance to include also GPs less interested in lifestyle counselling. However, GPs networks also attract specific characters, such as individuals particularly interested in sharing and learning from colleagues.

## Conclusion

This qualitative study provides detailed insights into German GPs’ knowledge of PA and conceptualisations of PA in CHD care, alongside their content, didactic, and organisational needs for training on delivering routine PA advice. Building on our prior analysis of GPs’ experiences and attitudes [34], these findings provide a foundation for developing tailored training aimed at positively influencing GPs’ practices in advising patients with CHD on PA.

Training aligned with identified needs and focused on brief advise is expected to enhance practical implementation and acceptance among GPs. We will evaluate its effectiveness in a subsequent study. If successful, the training could support the implementation of CHD guidelines regarding PA recommendations, improve primary care for patients with CHD, and strengthen PA referral schemes. Moreover, such training may be adaptable for other HPs, the management of other chronic conditions, and diverse healthcare settings.

## Supplementary Information

Supplementary Material 1.

Supplementary Material 2.

Supplementary Material 3.

## Data Availability

All data relevant to the study are included in the article or uploaded as online supplemental information.

## Abbreviations

CHD: Coronary heart disease
COREQ: Consolidated Criteria for Reporting Qualitative Research
DMP: Disease Management Programme
GP: General practitioner
HP: Healthcare professional
NRW: North-Rhine-Westphalia
OSF: Open Science Framework
PA: Physical Activity
WHO: World Health Organization
