# Supplementary material for: General practitioners’ educational and training needs and requirements for advising patients with coronary heart disease on physical activity: findings from a qualitative study in Germany
Source: BMC Prim Care. 2025 Aug 29;26:273. doi: 10.1186/s12875-025-02973-0 (PMC12395830; doi:10.1186/s12875-025-02973-0)
Supplement: Supplementary file 2 — Supplementary Material 2. [file 12875_2025_2973_MOESM2_ESM.pdf]

www.opticor-study.info

Version 3 (translated from German into English)

26.05.25

This work is licensed under the
Creative Commons Attribution

Work package 2 - Qualitative survey of General Practitioners (GPs)

Interview guide

Study goal

To gather general practitioners' (GPs) personal experiences and attitudes regarding advising patients with coronary heart disease (CHD) about physical activity. The aim is to identify motivating factors, barriers, and supportive conditions influencing such advice, and to derive both content-related and organisational needs for a training concept from the perspective of GPs.

Instructions and checklist for interviewers

- Review the participant's short questionnaire beforehand
- Conduct a technical check (volume, feedback, battery level)
- Prepare materials for taking notes and memos/postscript
- Ensure a quiet interview environment and avoid disturbances
- Take an open, inviting stance and use prompts that encourage storytelling (active listening!)
- The sub-questions in italics are examples for follow-ups – they do not all need to be asked
- Allow pauses and give interviewees time to reflect before answering

Greeting and introductory prompt

'Thank you for agreeing to participate in this interview as part of the OptiCor project, focusing on discussions between GPs and patients with coronary heart disease about physical activity.

I've prepared a few questions on this topic. As mentioned, the interview may take up to an hour, but often we finish earlier – it varies from case to case.

As discussed with me/my colleague during the consent process, I'd like to record the interview. You mentioned that this would be okay. I'd also like to take some notes. Could you please confirm again that you're fine with the recording and note-taking? That would really help me.

Your personal experiences and views are very important to us. Please take your time when answering and feel free to share anything that comes to mind. You're welcome to add anything else at any time. I also want to emphasize that there are no right or wrong answers. I'm only interested in your personal experiences and views. If there's a question you don't want to answer, that's perfectly fine – just let me know.

Finally, let me reassure you that everything you share will be treated confidentially, as outlined in the consent form.'

[Start recording]

1. Experiences with advice on physical activity in the context of CHD

1.1 Narrative question: Do you remember your last patient with coronary heart disease in your practice? Can you walk me through that consultation? Please describe in as much detail as possible how you experienced it.

When asked about the narrative prompt: *Whatever comes to mind spontaneously*
Ask when an acute case is reported: *Perhaps as part of a Disease Management Program (DMP)?*
Possible follow-up questions:

- *What approach did you take?*
- *What particularly stands out in your memory?*
- *What was unique about this patient?*
- *What's your usual routine in these situations?*
- *What other topics do you usually discuss in these sessions? Could you tell me about some other similar consultations?*

1.2 Narrative question: And how was it with those patients when it came to the topic of physical activity? Could you tell me more about that?

Possible follow-up questions:

- *How does the topic of physical activity usually come up in your consultations?*
- *What do you recommend to your patients?*
- *On average, how long do these conversations about physical activity take?*
- *What types of patients do you typically talk to about this?*
- *Based on your experience, how do patients generally respond when you bring up physical activity? Could you describe that in more detail?*
- *You see a wide variety of patients – how do the conversations differ among them?*
- *Every doctor has their own style – how would you describe yours when it comes to these conversations?*

If physical activity was not discussed: What do you think about the fact that it hasn't come up in those conversations so far?

2. Personal attitudes and motivators for physical activity advice

2.1 Narrative question: In your role as a GP, what do you see as the most important things you can do for patients with stable CHD?

Possible follow-up questions:

- *What are your main responsibilities in their care?*
- *What steps do you think are essential in providing care?*
- *Specifically regarding physical activity – what's your role in that?*
- *What personal meaning do these conversations about physical activity have for you?*
- *What kind of influence do you think you have on your patients when it comes to their activity levels?*

2.2 Narrative question: When you talk with patients who have CHD, what topics are important for you to cover?

Possible follow-up questions:
- *What do you wish for your CHD patients in general?*
- *What do you hope for them specifically in relation to physical activity?*
- *What do you focus on when discussing physical activity with them?*
- *What goals do you have in those conversations?*
- *What key messages do you aim to get across?*

2.3 Narrative question: What are your personal thoughts about physical activity for people with CHD?

Possible follow-up questions:
- *And how do you personally deal with physical activity in your own life?*

2.4 Narrative question: So far, we've talked about your experiences and priorities when it comes to these conversations. Now I'd like to ask: how does this play out in your daily practice? What's your experience with this in everyday routines?

Possible follow-up questions:
- *What aspects of it work well?*
- *Where do you still need support?*
- *What do you need to be able to offer these types of conversations?*
- *Do you have specific suggestions about what would help facilitate these discussions?*
- *And from a more organizational point of view – how do you manage this in your practice or as a team?*
- *What do you personally need to make these conversations happen?*
- *Thinking practically, what would help you in your day-to-day work when it comes to talking to CHD patients about physical activity?*
- *You've been doing this for a while now – what do you think would help younger colleagues just starting out?*
- *Final question (ask last in this section): You've had quite a bit of experience with this. What do you think would help younger colleagues who are new to the field?*

3. Requirements for a training programme

3.1 Narrative question: Now that we're talking about training – I'd like you to imagine a professional development course or training for GPs on advising patients with CHD about physical activity. What comes to mind?

Possible follow-up questions:
- *What components of such a course would personally help you?*
- *In your opinion, what essential or important content should such training for GPs include?*
- *What should definitely not be part of it?*
- *What would a course have to look like for you to want to attend?*
- *Is there anything that might keep you from participating in such training?*
- *How should the content be delivered? What would your preferences be?*
- *I'd like to ask specifically about your preferences regarding the length of the course, suitable days, and location. What are your requirements there?*
- *What do you think would get your colleagues excited about participating?*

3.2 Narrative question: Can you recall a training (or several) that you left feeling truly inspired? Feel free to tell me more about it.

Possible follow-up questions:

- *What were your expectations going in, and how were they met or not met?*
- *What made this training stand out?*
- *On the other hand, are there any trainings you remember negatively? What made them disappointing?*

4. Final thoughts and anything left unsay

4.1 Narrative question: We're now at the final question. Thank you – your insights have been incredibly valuable. If you could wish for three things related to what we've discussed in the past hour – what would they be?

Possible follow-up questions:

- *Is there anything else you'd like to add?*
- *Was there something important that didn't get mentioned?*

Thank you very much for your time and your willingness to participate in this interview.

Memo Sheet

**Pseudonym**	
**Date/Time/Duration of the interview**	
**Date/Time memo was created**	

Where did the interview take place?

External conditions and disruptions before, during, or after the interview (e.g., distractions from phone calls, interruptions, relaxed atmosphere, long waiting times, early termination, etc.)

How did the interviewee seem? (e.g., mood, interest, motivation)

Interviewer's own feelings, mood, and associations during the interview

Interview flow/dynamics

Notable topics: Covered and missing themes – especially those relevant to the research questions or that go beyond them

Other observations/comments (What stands out? What's relevant for the project?)
