# Supplementary material for: General practitioners’ educational and training needs and requirements for advising patients with coronary heart disease on physical activity: findings from a qualitative study in Germany
Source: BMC Prim Care. 2025 Aug 29;26:273. doi: 10.1186/s12875-025-02973-0 (PMC12395830; doi:10.1186/s12875-025-02973-0)
Supplement: Supplementary file 3 — Supplementary Material 3. [file 12875_2025_2973_MOESM3_ESM.pdf]

www.opticor-study.info

Version 5 (translated from German into English)

26.05.25

This work is licensed under the Creative Commons Attribution

Work package 2 - Qualitative survey of General Practitioners (GPs)

Focus group guide

Study goal

To gather general practitioners' personal experiences and perspectives on advising patients with coronary heart disease (CHD) regarding physical activity. The aim is to identify motivators, barriers, and supportive factors that affect these conversations. Additionally, the group discussion will help define both content-related and organizational requirements for a training concept from a GP's perspective.

Another key objective is to explore the group's interest in participating in a pilot training and accompanying evaluation.

Instructions and checklist for moderators

- Review participants' short questionnaires in advance
- Conduct a technical check (volume, feedback, battery level)
- Ensure a calm and quiet discussion environment
- Have materials ready for note-taking and memos
- Allow for pauses—give participants time to think
- Moderators should speak minimally—let participants talk
- Record seating positions and prepare a discussion protocol

1) Opening phase and welcome

'Thank you very much for agreeing to take part in this group discussion.

My name is ____, I work as a ____ at the Institute of General Practice at the University of Düsseldorf, and I will be moderating today's session.

Joining me is ___, who will support us by documenting the discussion.

Our topic is one that lies at the heart of general practice, which is why your insights and experience are especially important to us.

This session is about your day-to-day realities, your expert assessments, and your practical suggestions. How do you experience consultations with your patients, and what changes do you think are feasible?’

Audio recording, protocol and confidentiality

‘As mentioned before, everything shared here today will be kept confidential. My colleague will be taking notes. Please don’t be distracted if I write something down as well—it just helps me keep track and come back to things if needed.

This session will be audio recorded, as previously discussed. I’ll turn the recorder on shortly.

Please try to ensure that only one person speaks at a time. Every viewpoint matters and should be heard. It would help if you place your phone away from the recorder—or ideally switch it off or at least put it in airplane mode to avoid interference.

I also want to make a mutual agreement that everything said here stays in this room. Please don’t share what others have said outside the group.

Throughout the session, I’ll ask you a series of open-ended questions. I encourage you to discuss whatever seems important to you. There are no right or wrong answers, and I won’t be evaluating your comments.’

Brief project overview

‘Before we begin, let me quickly recap the purpose of today’s discussion. The OptiCor project focuses on conversations between GPs and their patients with coronary heart disease about physical activity.

Do you have any general questions before we get started?

If not, I’ll now turn on the recorder.’

[Start recording]

Participant introductions (optional)

‘Let’s start with a quick round of introductions. Please briefly introduce yourselves.’

2) Topic 1 – Needs and requirements for advice on physical activity in CHD care

<table>
<tr><th colspan="3">Narrative question:</th></tr>
<tr><td colspan="3">You likely care for patients with coronary heart disease in your practice. When you talk to these patients, what do you usually discuss? Try to recall a recent consultation—maybe from the past few days or weeks—perhaps within the CHD Disease Management Programme.</td></tr>
<tr><th>Key themes to explore</th><th>Follow-up questions to deepen the discussion</th><th>Specific questions</th></tr>
<tr>
<td>- Actual experiences and observations from practice (status quo)
- Conditions required to offer physical activity advice
- Needs and expectations from the GP’s point of view
- Typical content of conversations about physical activity
- Assumptions about patients’ expectations or wishes
- What makes a conversation “good”, in general and specifically around physical activity
- Attitudes and opinions about GP counseling on physical activity in the context of CHD</td>
<td><u>Explanation</u>:
- Could you elaborate a bit more on that?
- What exactly did you mean by that?
- Can you give a specific example?
- Do you have a situation in mind you’d like to share?
- What exactly do you mean by that—would you say that’s positive or negative?
<u>If repetition is needed:</u>
- I didn’t quite catch that—could you repeat it?
<u>To encourage further input:</u>
- Is there anything else that comes to mind?
- What other thoughts do you associate with this topic?
- What does this topic make you think of?
<u>To invite different perspectives</u>
- Maybe there are different views on this—what do others think?
- Has anyone had a different experience?</td>
<td>Always with indexicality (=subjective meaning of an expression. Is it meant positively or negatively etc.), what does that mean (exactly)?

- What is important to you when discussing physical activity with patients who have CHD?
- From your perspective, what makes for a ‘good conversation’ with a patient?
- What kind of content must be included in such a conversation, in your opinion?
- What would help make these conversations enjoyable or fulfilling for you (again)?
- What support do you need to be able to conduct these discussions with your CHD patients?
- Based on your experience, how do patients generally respond to these conversations?
- What do you think it takes for patients to take your advice seriously and act on it?</td>
</tr>
</table>

3) Topic 2 – Requirements for a training programme

<table>
<tr><td colspan="3">Erzählaufforderung:</td></tr>
<tr><td colspan="3">Note: Begin this section by briefly summarizing the issues and challenges raised in Topic 1, to transition naturally into the discussion about training.

I'd like to invite you to imagine a training session on how to talk with patients about physical activity. What would you need for it to be helpful? What comes to mind?

<u>To encourage further input:</u> What would the training need to include for you to leave feeling equipped and motivated to hold effective and enjoyable conversations about physical activity the very next day?</td></tr>
<tr><th>Key themes to explore</th><th>Follow-up questions to deepen the discussion</th><th>Specific questions</th></tr>
<tr><td>- Expectations for a GP training programme on conversations with patients with CHD
- Ideas and preferences regarding content and structure of the training
- Prerequisites and conditions for participation
- Didactic preferences (e.g. teaching format, learning methods)</td><td><u>Explanation</u>:
- Could you elaborate a bit more on that?
- What exactly did you mean by that?
- Can you give a specific example?
- Do you have a situation in mind you'd like to share?
- What exactly do you mean by that—would you say that's positive or negative?
<u>If repetition is needed:</u>
- I didn't quite catch that—could you repeat it?
<u>To encourage further input:</u>
- Is there anything else that comes to mind?
- What other thoughts do you associate with this topic?
- What does this topic make you think of?
<u>To invite different perspectives</u>
- Maybe there are different views on this—what do others think?
- Has anyone had a different experience?</td><td>Always with indexicality (=subjective meaning of an expression. Is it meant positively or negatively etc.), what does that mean (exactly)?

- Do you have any ideas about what a training for GPs on advising patients with CHD about physical activity could look like — one that would truly inspire and engage you?
- In your opinion, what are the key topics or elements that must be included?
- What would make such a course appealing enough for you to participate?
- Is there anything that would discourage you from attending?
- How should the content be delivered—what would your ideal format be?
- What do you think would get your colleagues interested in joining?</td></tr>
</table>

4) Final question

‘At the end of this training on advising patients about physical activity—what would need to happen during that session for you to feel ready and motivated to go back and have good conversations with your CHD patients the next day?’

5) Closing phase and farewell

‘That brings us to the end of our session. We’ve touched on many aspects today, but surely there are still topics that haven’t come up

Is there anything else you’d like to share—anything important to you that we haven’t discussed yet? Did we miss something that should be mentioned?’

Closing thanks

‘I’d like to sincerely thank you for your participation and for such a lively and valuable discussion.’

Tasks for the study team

- Responsible for setup and technical equipment
- Provide participants with food and drinks
- Arrange chairs and tables to avoid distractions
- Greet and brief participants; manage logistics
- Create a seating diagram with numbers: 3

  2 4

  1 5
- Diagram should also include microphone placement, participant positions (numbered clockwise as A, B, C or 1, 2, 3), and gender noted as m or f
- Help identify speakers for transcription purposes (note speech characteristics like volume, stammering, pitch, or interaction patterns)
- Ensure backup batteries, a second recording device, and sufficient memory (devices stop recording when full!)

Memobogen

**Pseudonym**	
**Date/Time/Duration**	
**Date/Time memo was created**	

Location of the session?

Atmosphere (location, mood, participants’ behavior, non-verbal impressions)

How did the participants seem? (e.g., mood, interest, motivation)

Own impressions and emotions as moderator

Group dynamics and relationships among participants

Discussion flow and development

Noteworthy interactions

Key themes touched upon or omitted (relevant to the study or beyond)

Any additional comments or observations relevant to the project

External conditions or disruptions before, during, or after the session (e.g., calls, delays, interruptions)
